# Correlation between ultrasonographic measurements of the ileum and serum cobalamin concentrations in healthy cats and cats with chronic gastrointestinal signs: A pilot study

**DOI:** 10.29374/2527-2179.bjvm000626

**Published:** 2026-06-26

**Authors:** Iago Martins Oliveira, Letícia Oliveira Silva, Maria Eduarda Cardoso Cysneiros, Vitor Guimarães Ribeiro, Maria Fernanda Cavalcante Ramos

**Affiliations:** 1 Escola de Ciências Médicas e da Vida, Pontifícia Universidade Católica de Goiás, Goiânia, GO, Brazil.

**Keywords:** biomarker, enteropathy, feline, intestine, ultrasonography, biomarcador, enteropatia, felino, intestino, ultrassonografia

## Abstract

Assessing intestinal health in cats with chronic gastrointestinal signs remains challenging. Serum cobalamin concentration is commonly used as a marker of ileal dysfunction, whereas ultrasonography enables morphological evaluation of the gastrointestinal tract. This study investigated the correlation between ultrasonographic ileal wall thickness and serum cobalamin concentrations in healthy cats and cats with chronic gastrointestinal signs. Twelve cats were divided into a symptomatic group (G1, n = 6) and a healthy control group (G2, n = 6) and underwent laboratory testing and abdominal ultrasonography. Mean ileal thickness was slightly greater in G1 (0.22 ± 0.03 cm) than in G2 (0.21 ± 0.02 cm), although the difference was not statistically significant (p = 0.48). Serum cobalamin concentrations also did not differ significantly between groups (p = 0.81). Correlation analysis demonstrated a weak, nonsignificant positive correlation in G1 and a moderate, nonsignificant correlation in G2. These findings suggest that ileal thickness alone is not a reliable predictor of serum cobalamin concentration and highlight the need for multifactorial diagnostic approaches and studies with larger sample sizes.

## Resumo

A avaliação da saúde intestinal em gatos com sinais gastrointestinais crônicos permanece um desafio. A cobalamina sérica é comumente utilizada como marcador de disfunção ileal, enquanto a ultrassonografia permite a avaliação morfológica do trato gastrointestinal. Este estudo investigou a correlação entre a espessura ileal ultrassonográfica e as concentrações séricas de cobalamina em gatos saudáveis e gatos com sinais gastrointestinais crônicos. Doze gatos foram divididos em grupos sintomáticos (G1, n = 6) e saudáveis (G2, n = 6) e submetidos a exames laboratoriais e ultrassonografia abdominal. A espessura ileal média foi ligeiramente maior no G1 (0,22 ± 0,03 cm) do que no G2 (0,21 ± 0,02 cm), sem significância estatística (p = 0,48). As concentrações séricas de cobalamina não diferiram entre os grupos (p = 0,81). A análise de correlação revelou uma correlação positiva fraca e não significativa no G1 e uma correlação moderada e não significativa no G2. Esses resultados indicam que a espessura do íleo isoladamente não é um preditor confiável dos níveis séricos de cobalamina, destacando a necessidade de abordagens diagnósticas multifatoriais e estudos mais amplos.

## Introduction

Chronic feline enteropathy is characterized by gastrointestinal signs persisting for 3 weeks or longer. The most common clinical signs include vomiting, diarrhea, weight loss, hyporexia, and anorexia. Definitive diagnosis requires exclusion of infectious, parasitic, hepatic, renal, and metabolic diseases, in addition to intestinal biopsy, histopathological evaluation, and immunohistochemistry (Marsilio, 2021). Although any intestinal segment may be affected, the ileum is frequently involved, particularly in enteropathy associated with lymphocytic lymphoma (Moore et al., 2012).

Laboratory and imaging examinations are valuable for identifying and characterizing intestinal lesions in cats with enteropathy (Marsilio, 2021). Abdominal ultrasonography is an important diagnostic tool because it enables topographic assessment, evaluation of intestinal wall stratification, analysis of peristalsis, and visualization of abdominal lymph nodes and adjacent organs, including the liver, pancreas, and gallbladder. Diffuse thickening of the mucosal, submucosal, or muscular layers is the most commonly reported ultrasonographic finding in cats with chronic gastrointestinal inflammation (Marsilio et al., 2023). A recent study further emphasized the importance of ultrasonography in the evaluation of feline enteropathy, although it also highlighted its limitations in establishing a definitive diagnosis (Beatrice et al., 2025).

Among the serum biomarkers used in the diagnosis of feline intestinal diseases, cobalamin (vitamin B12), which is absorbed in the ileum, is of particular interest (Ruaux, 2013). Hypocobalaminemia has frequently been associated with severe ileal inflammation, liver disease, exocrine pancreatic insufficiency, and dysbiosis (Riggers et al., 2022; Siani et al., 2023). Unlike dogs and humans, few studies have linked cobalamin deficiency to poor prognosis in cats with gastrointestinal disease. Nevertheless, a recent study demonstrated an association between hypocobalaminemia and reduced survival in this species (Borgonovi & Bayton, 2025).

We hypothesized that cats with chronic gastrointestinal signs would exhibit greater ultrasonographic ileal thickening and that this alteration would be associated with lower serum cobalamin concentrations. Therefore, this study aimed to evaluate the correlation between ultrasonographic ileal measurements and serum cobalamin concentrations in cats with chronic gastrointestinal signs and to compare these findings with those obtained from healthy cats.

## Material and methods

The study protocol was approved by the Animal Ethics Committee of the Pontifical Catholic University of Goiás (PUC Goiás) under protocol number 4407250821. The study population consisted of 12 domestic cats treated at a university veterinary hospital. All cats underwent the same clinical and laboratory screening procedures, including general and gastrointestinal-specific physical examinations, complete blood count, serum biochemistry analyses [gamma-glutamyltransferase (GGT), alanine aminotransferase (ALT), creatinine, urea, and albumin], serum cobalamin and folate measurements, rapid testing for feline immunodeficiency virus (FIV) and feline leukemia virus (FeLV; SNAP FIV/FeLV Combo, Idexx, São Paulo, SP, Brazil), and polymerase chain reaction (PCR) testing for viral RNA and proviral DNA of FIV and FeLV, in addition to total abdominal ultrasonography. Participation in the study required written informed consent from the owners.

The cats were allocated into two experimental groups: G1, comprising six cats with chronic gastrointestinal signs, and G2, comprising six clinically healthy cats. Inclusion criteria for G1 were cats of either sex, neutered, mixed breed, aged between 6 months and 13 yr, and weighing between 1 and 7 kg. Cats presenting chronic gastrointestinal signs, including vomiting and/or diarrhea, persisting for >4 weeks, were eligible for inclusion. Etiological diagnosis was not mandatory for enrollment; therefore, inclusion was based solely on the presence and chronicity of clinical signs. In addition, cats in the symptomatic group underwent a 60-day dietary trial using a hypoallergenic hydrolyzed-protein diet. Parasitic infections were excluded through three coproparasitological examinations of fresh fecal samples, qualitative PCR for Tritrichomonas blagburni, and immunochromatographic testing for Giardia duodenalis (Conclue Giardiase, Ourofino, São Paulo, SP, Brazil). Total thyroxine (T4) concentrations were measured by radioimmunoassay, and feline-specific pancreatic lipase and trypsinogen concentrations were also evaluated in this group.

The inclusion criteria for G2 included the same species, age range, body weight range, sex, breed, and reproductive status as those established for G1. However, cats in this group were required to have no history of disease or clinical signs. In addition to normal findings on physical examination, all cats in G2 were required to present normal hematological, biochemical, and B-mode abdominal ultrasonographic parameters. Cats with abnormal clinical, laboratory, or ultrasonographic findings, according to species-specific reference values, were excluded. These preliminary evaluations were performed to confirm the health status of the control group.

All cats underwent food fasting for 8 h and water restriction for 2 h before ultrasonographic examination. Abdominal ultrasonography was performed using a SAEVO FT422 ultrasound system equipped with microconvex and linear transducers operating at frequencies between 7.5 and 10 MHz, adjusted according to the animal’s body condition. Cats were positioned in dorsal recumbency, and a wide abdominal trichotomy was performed before application of acoustic coupling gel to optimize transducer–skin contact.

A systematic abdominal ultrasonographic examination was performed to evaluate the size, shape, contour, echogenicity, and echotexture of abdominal organs. The examination followed a standardized scanning protocol, including assessment of the gastrointestinal tract in both longitudinal and transverse planes.

Focused evaluation of the gastrointestinal tract prioritized assessment of the ileum, including its anatomical location, wall thickness, stratification pattern, echogenicity, and peristalsis. The intestinal wall was identified based on its characteristic five-layer stratification pattern. Wall thickness was measured in centimeters using electronic calipers integrated into the ultrasound software. Measurements were obtained from the serosal to the mucosal interface in transverse images while avoiding areas affected by luminal distension or compression artifacts.

All ultrasonographic examinations were performed and interpreted by the same experienced operator, who was blinded to group allocation to minimize measurement bias.

According to Griffin (2019), normal small-intestinal wall thickness in cats ranges from approximately 0.20 to 0.28 cm, with preservation of normal wall layering. These reference values were used to interpret the ultrasonographic findings of the present study.

Data were analyzed using descriptive statistics and expressed as mean, standard deviation, and coefficient of variation. Data normality was assessed using the Shapiro–Wilk test. Parametric and unpaired variables were analyzed using Welch’s corrected t test. Pearson’s correlation coefficient was used to assess correlations between variables. Correlation strength was interpreted as follows: 0.00–0.19, very weak; 0.20–0.39, weak; 0.40–0.69, moderate; 0.70–0.89, strong; and ≥0.90, very strong (Mukaka, 2012). Differences were considered statistically significant at p < 0.05. Statistical analyses and graph generation were performed using GraphPad Prism 10.

## Results

**Figure 1:**
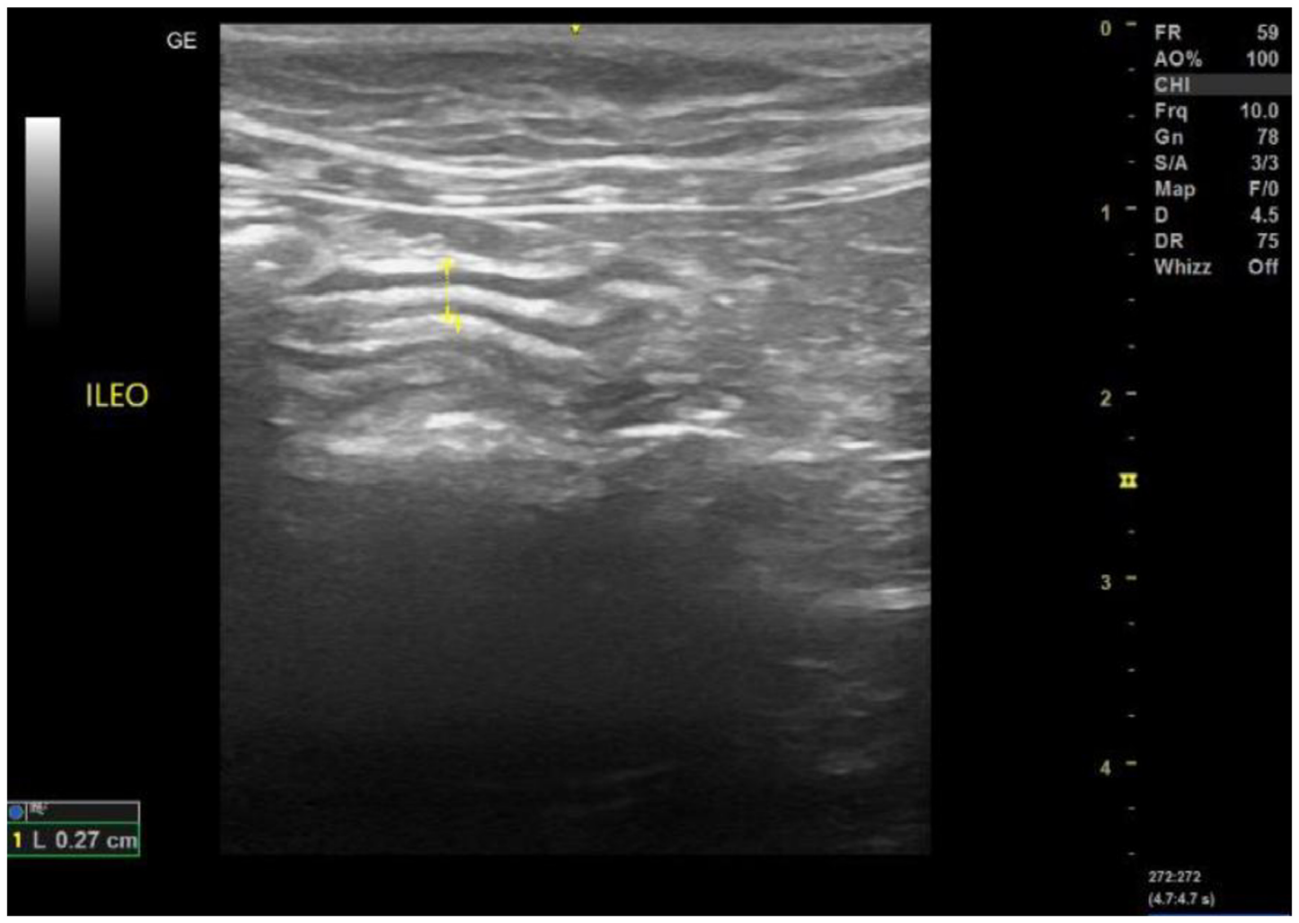
Representative B-mode ultrasonographic image of the feline ileum obtained using a SAEVO FT422 ultrasound system equipped with a high-frequency linear transducer (7.5 MHz). The image was acquired in a transverse plane, allowing clear visualization of the normal intestinal wall stratification pattern. Ileal wall thickness was measured using electronic calipers integrated into the ultrasound software, with measurements taken from the outer serosal surface to the luminal mucosal interface.

Descriptive statistics for the evaluated parameters in cats from G1 and G2 are presented in Table 1. Representative ultrasonographic images illustrating ileal wall measurement and preservation of normal intestinal wall stratification are shown in Figure 1.

**Table 1:** Mean, standard deviation, confidence interval, and coefficient of variation for body weight, age, red blood cells, platelets, total leukocytes, total plasma proteins, alanine aminotransferase, creatinine, and intestinal wall thickness (duodenum, jejunum, ileum, and colon), as well as serum cobalamin and folate concentrations in cats with chronic gastrointestinal signs (G1) and healthy cats (G2).

**G1 – cats with chronic gastrointestinal signs**					
**Variable**	**Mean/Unit of measure**	**Standard Deviation**	**Mean CI (95%) low**	**Mean CI (95%) high**	**Coefficient of variation (%)**
Weight	4.16 kg	0.72	3.40	4.92	17.35
Age	4.1 years	3.54	0.44	7.88	85.08
RBC	9.21 x10^6^/ μL	1.25	7.89	10.53	13.59
Platelets	285.3 x10^3^/ μL	44.28	238.86	331.80	15.52
WBC	8633 / μL	1056	7525	9741	12.23
TPP	8.3 g/dL	0.81	7.47	9.1	9.78
ALT	67.18 U/L	25.07	40.87	93.50	37.32
Creatinine	1.41 mg/dL	0.25	1.14	1.68	18.03
Duodenum	0.21 cm	0.02	0.19	0.24	11.46
Jejunum	0.19 cm	0.02	0.16	0.22	14.20
Ileum	0.22 cm	0.03	0.19	0.25	13.69
Colon	0.13 cm	0.03	0.10	0.17	25.29
Cobalamin	810.7 pg/mL	277.9	519	1102	34.28
Folate	7.38 ng/mL	4.59	2.55	12.21	62.28
**G2 – healthy cats**					
Weight	5.0 kg	1.26	3.67	6.32	25.30
Age	5.8 years	2.13	3.59	8.07	36.63
RBC	8.46 x10^6^/ μL	1.19	7.21	9.71	14.06
Platelets	287.7 x10^3^/ μL	44.23	241.5	334.3	15.36
WBC	7455 / μL	1109	6291	8618	14.87
TPP	6.66 g/dL	1.03	5.58	7.75	15.49
ALT	62.25 U/L	20.71	40.52	83.98	33.26
Creatinine	1.17 mg/dL	0.26	0.89	1.45	22.92
Duodenum	0.21 cm	0.03	0.17	0.24	17.27
Jejunum	0.20 cm	0.02	0.18	0.23	13.04
Ileum	0.21 cm	0.02	0.18	0.23	10.56
Colon	0.14 cm	0.03	0.1	0.17	21.65
Cobalamin	847.0 pg/mL	255.7	578.7	1115	30.19
Folate	6.06 ng/mL	0.56	5.47	6.60	9.31

*Note*: TPP = Total plasma proteins; ALT = Alanine aminotransferase; RBC = Red blood cells; WBC = White Blood Cells.

In G1, the mean ultrasonographic ileal wall thickness was 0.22 ± 0.03 cm, with a 95% confidence interval (95% CI) ranging from 0.19 to 0.25 cm. In G2, the mean ileal wall thickness was 0.21 ± 0.02 cm, with a 95% CI ranging from 0.18 to 0.23 cm. No statistically significant difference in ileal thickness was observed between groups (p = 0.48).

Regarding serum cobalamin concentrations, cats in G1 presented a mean value of 810.7 ± 277.9 pg/mL, with a 95% CI ranging from 519 to 1,102 pg/mL, whereas cats in G2 showed a mean concentration of 847.0 ± 255.7 pg/mL, with a 95% CI ranging from 578.7 to 1,115 pg/mL. Similarly, no statistically significant difference in serum cobalamin concentration was detected between groups (p = 0.81; Figures 22B).

**Figure 2:**
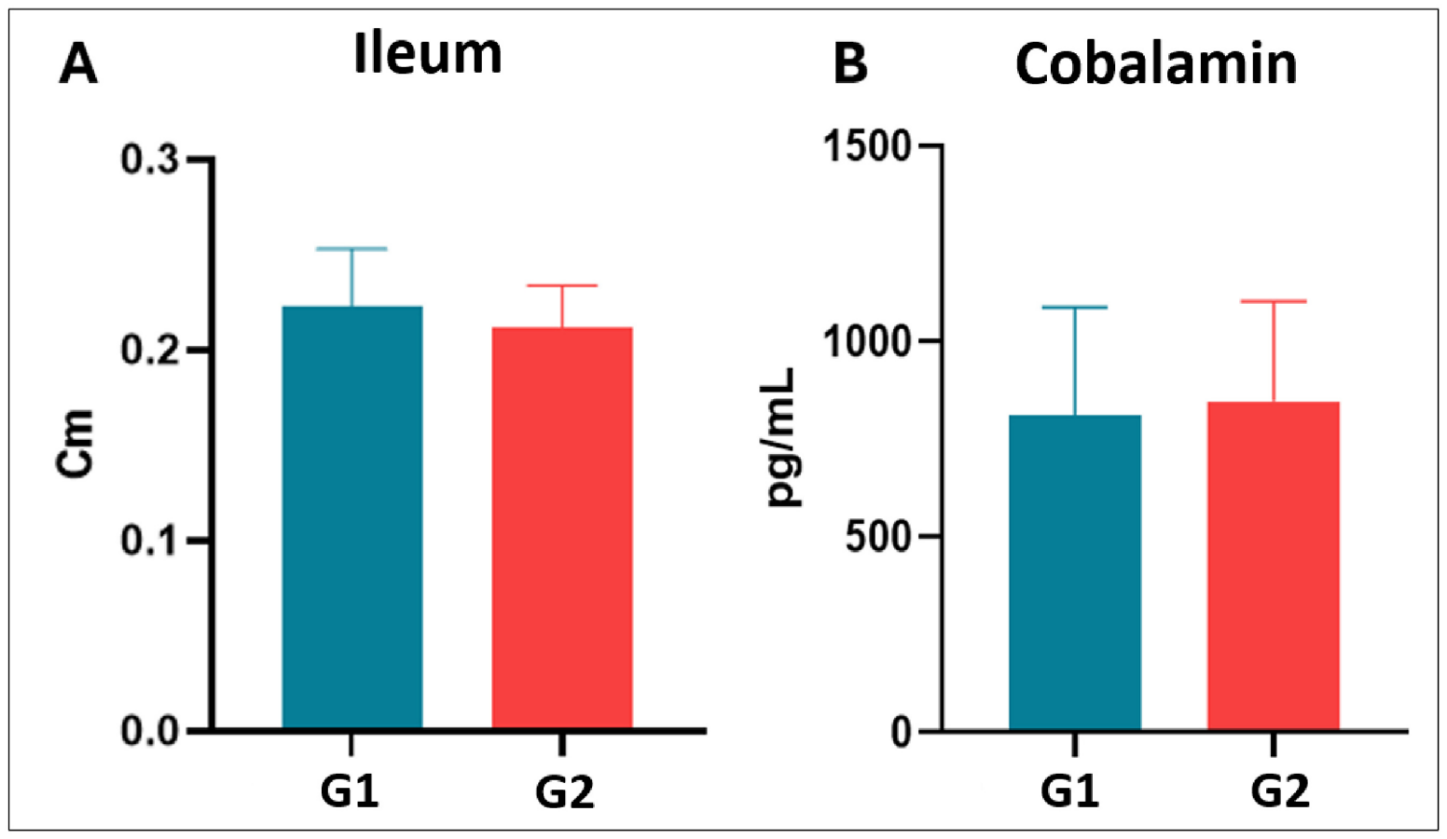
Mean ± standard deviation values for ileal wall thickness and serum cobalamin concentrations in cats with chronic gastrointestinal signs (G1) and clinically healthy cats (G2). (A) Comparison of ultrasonographic ileal wall thickness between groups; (B) Comparison of serum cobalamin concentrations between groups. Variables that met the assumptions of normality according to the Shapiro–Wilk test were analyzed using Welch’s t test, with statistical significance set at p < 0.05.

**Figure 3:**
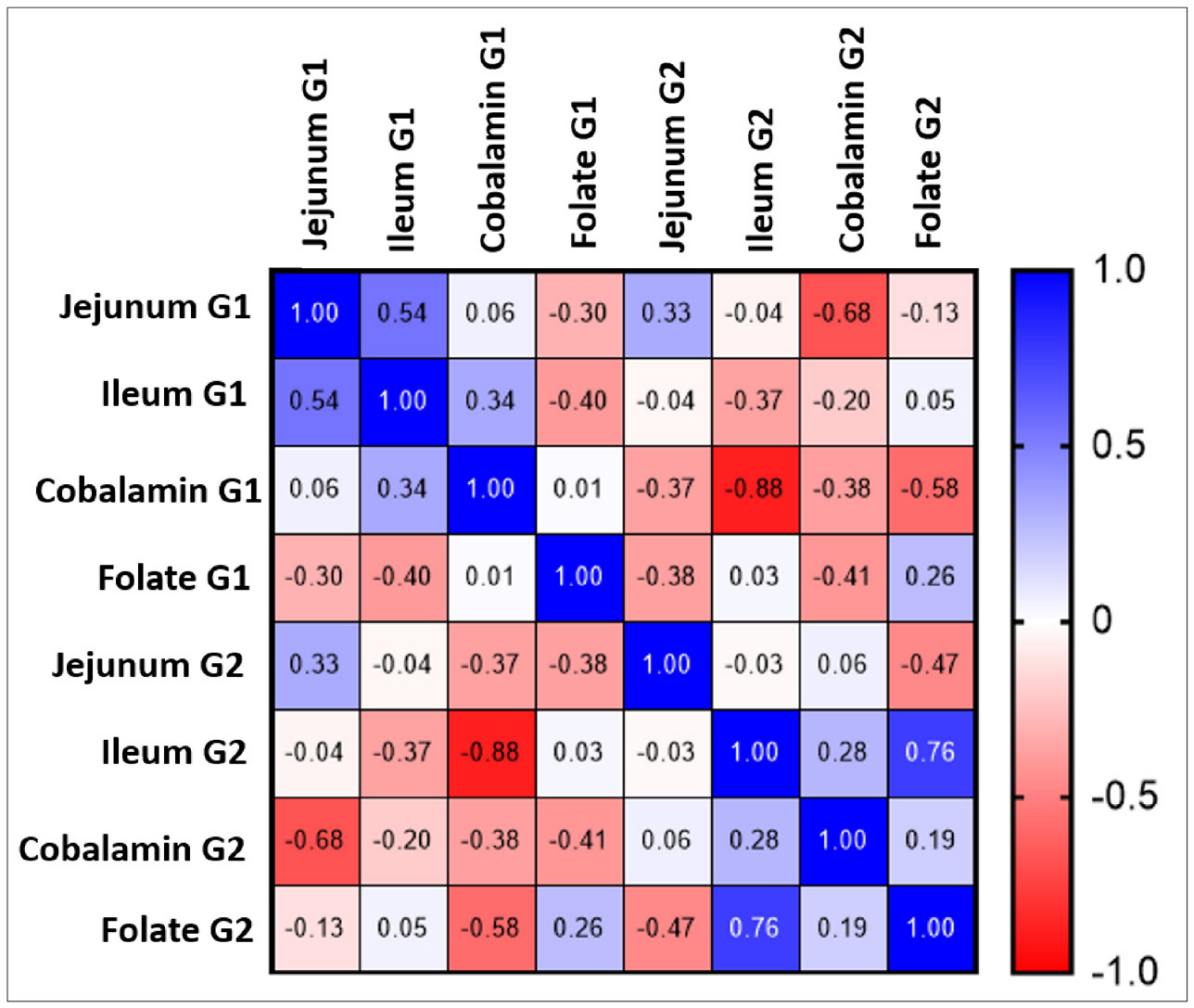
Correlation matrix illustrating the relationship between serum cobalamin concentrations and ultrasonographic ileal wall thickness in cats with chronic gastrointestinal signs (G1) and healthy control cats (G2). Weak to moderate positive correlations were identified between the evaluated variables; however, none of the observed associations reached statistical significance (p > 0.05).

Pearson’s correlation analysis was performed to evaluate the relationship between serum cobalamin concentrations and ultrasonographic ileal wall thickness (Figure 3). In G1, a weak positive correlation was observed between ileal wall thickness and serum cobalamin concentration (r = 0.056), indicating a minimal association between increased ileal thickness and higher cobalamin concentrations. However, this correlation was not statistically significant (p = 0.92). In G2, a moderate positive correlation was identified between these variables (r = 0.284), suggesting a possible association between increased ileal thickness and higher serum cobalamin concentrations. Nevertheless, this correlation was also not statistically significant (p = 0.59).

## Discussion

Cats in G1 showed gastrointestinal signs for at least 4 weeks and were therefore clinically classified as having chronic enteropathy. This classification is consistent with the definition proposed by Marsilio et al. (2023), who established that gastrointestinal signs must persist for at least 3 weeks. Although the cats presented similar clinical signs, the etiological diagnosis was not established because intestinal biopsies, histopathology, and immunohistochemistry were not performed. Despite the lack of a definitive diagnosis, all recommended pre-intestinal biopsy screening procedures were performed according to current literature recommendations (Drut et al., 2024; Marsilio et al., 2023; Yu et al., 2023), including exclusion of parasitic, infectious, systemic, and food-responsive diseases. Although this represents a limitation of the present study, we believe that these preliminary findings may contribute to future studies involving more clearly defined groups.

Symptomatic cats showed higher mean values and standard deviations for ultrasonographic ileal measurements than cats in the healthy control group. This finding is consistent with previous studies reporting increased intestinal wall thickness in cats with inflammatory and neoplastic enteropathies (Beatrice et al., 2025). However, unlike the aforementioned study, which demonstrated statistically significant differences and included histopathological confirmation, no significant differences were observed in the present study. This discrepancy may be related to the small sample size and the lack of a definitive etiological diagnosis, which may have limited the detection of more pronounced structural alterations.

Statistical analysis showed that symptomatic cats had lower mean serum cobalamin concentrations than healthy cats, consistent with previous reports (Jugan & August, 2017; Maunder et al., 2012; Ruaux, 2013), which describe hypocobalaminemia as a common finding in chronic enteropathies due to ileal malabsorption, impaired interaction with the cobalamin–intrinsic factor complex, and intestinal dysbiosis. However, dysbiosis was not evaluated in the present study. We believe that inclusion of the dysbiosis index proposed by Sung et al. (2022), together with correlations between serum cobalamin concentrations and ultrasonographic ileal measurements, would be important to assess the influence of dysbiosis on serum cobalamin reduction.

The present study found no statistically significant correlation between ileal thickness and serum cobalamin concentration in either the healthy or symptomatic groups (r = 0.056 and r = 0.284, respectively). These results suggest that ultrasonographic measurement of ileal thickness alone may not be a reliable predictor of serum cobalamin concentration. This lack of association indicates that structural changes identified by ultrasonography may not directly reflect functional impairment of cobalamin absorption, supporting previous observations that imaging findings and biochemical markers may represent different aspects of intestinal disease (Larson & Biller, 2009). Therefore, these parameters should be interpreted as complementary rather than isolated findings.

A major limitation of the present study is the small sample size, which may have limited the statistical power necessary to identify significant differences and correlations between the evaluated variables. Consequently, the lack of statistically significant associations should be interpreted cautiously, as it does not rule out the possibility of biologically meaningful relationships between ultrasonographic ileal measurements and serum cobalamin concentrations. Therefore, the findings reported here should be regarded as preliminary. Further investigations involving larger and more homogeneous populations are required to confirm and expand upon these observations.

## Conclusion

The findings of this study indicate that isolated measurement of ileal thickness by B-mode ultrasonography is not a reliable marker of ileal function with respect to cobalamin absorption. Therefore, integration of clinical, laboratory, and imaging findings is necessary for a more accurate diagnosis of feline enteropathies.
